# Medicalization of global health 3: the medicalization of the non-communicable diseases agenda

**DOI:** 10.3402/gha.v7.24002

**Published:** 2014-05-16

**Authors:** Jocalyn Clark

**Affiliations:** 1 icddr,b, Dhaka, Bangladesh; 2 Department of Medicine, University of Toronto, Toronto, Canada

**Keywords:** global health, non-communicable disease, chronic disease, medicalization, sociology

## Abstract

There is growing recognition of the massive global burden of non-communicable diseases (NCDs) due to their prevalence, projected social and economic costs, and traditional neglect compared to infectious disease. The 2011 UN Summit, WHO 25×25 targets, and support of major medical and advocacy organisations have propelled prominence of NCDs on the global health agenda. NCDs are by definition ‘diseases’ so already medicalized. But their social drivers and impacts are acknowledged, which demand a broad, whole-of-society approach. However, while both individual- and population-level targets are identified in the current NCD action plans, most recommended strategies tend towards the individualistic approach and do not address root causes of the NCD problem. These so-called population strategies risk being reduced to expectations of individual and behavioural change, which may have limited success and impact and deflect attention away from government policies or regulation of industry. Industry involvement in NCD agenda-setting props up a medicalized approach to NCDs: food and drink companies favour focus on individual choice and responsibility, and pharmaceutical and device companies favour calls for expanded access to medicines and treatment coverage. Current NCD framing creates expanded roles for physicians, healthcare workers, medicines and medical monitoring. The professional rather than the patient view dominates the NCD agenda and there is a lack of a broad, engaged, and independent NGO community. The challenge and opportunity lie in defining priorities and developing strategies that go beyond a narrow medicalized framing of the NCD problem and its solutions.

Non-communicable diseases (NCDs) have emerged as a substantial concern within the global health community due to their growing prevalence, the projected costs to societies of their impact and treatment, and recognition that they have traditionally been ignored relative to infectious disease in both public attention and donor funding. More than 60% of all deaths are said to be due to NCDs, amounting to 35 million people each year (1), and low- and middle-income countries (LMICs) bear 80% of this mortality burden (2). Declines in NCD deaths in high-income countries notwithstanding, there is growing concern that the epidemiological transition is rapidly creating a double burden of infectious and non-infectious diseases among people in LMICs. New global estimates suggest that NCDs will cost a staggering US$47 trillion by 2030 (3). And yet NCDs were excluded from the Millennium Development Goals programme and currently receive less than 3% of global health aid (4), factors that fuel their current advocacy.

With appreciation that NCDs have social, economic and environmental consequences threatening global development (5), the problem is now positioned as a ‘slow motion catastrophe’ (6, 7). Nascent recognition and advocacy were rewarded with a UN Summit on NCDs at the General Assembly in 2011 (only the second time in history that such an event focused on a health issue), and efforts since then to press the importance of the NCD issue and hold governments to their commitments in the 2011 political declaration (8) have involved a number of organised groups like the NCD Alliance (9), Lancet NCD Action Group (10) and the United Health Chronic Disease initiative (11), among others. The subsequent ‘25×25’ goal (25% reduction in premature NCD deaths by 2025) agreed by 194 member states at the 2012 world health assembly and the 2013 comprehensive action plan (12) committed governments to a set of actions to prevent and treat NCDs. The accompanying global monitoring framework is now being debated and consolidated. These shifts have enabled four ‘priority’ NCDs – cardiovascular disease, common cancers, chronic respiratory disease and diabetes, which share common risk factors such as unhealthy diet, physical inactivity, harmful use of alcohol and smoking – to rise in prominence on the global health agenda, and they now occupy an unprecedented place on the broader development agenda for post-2015 (13–15).

Current global targets involve a range of health outcomes (NCD mortality and morbidity), risk factor modification (reductions in physical inactivity, obesity, salt intake, blood pressure, tobacco use and harmful use of alcohol) and treatment (access to medicines and technologies, and drug therapy counselling). While much of the advocacy and evidence reviews that formed the basis for the rise of recognition of NCDs acknowledge their social determinants and emphasise the need for governments to adopt a ‘whole of society’ view and a multi-sectoral approach to meet targets (16), it appears clear that continued efforts are needed to ensure that action extends beyond a narrow medicalized response to NCDs.

A medicalization lens as described in the previous papers (17, 18) is helpful here because it reveals that the priorities advanced by various groups reflect differing values placed upon the role of individuals, the health system and government, the responsibilities of the private sector including industry and broader political action. As I have described in-depth in Paper 1 of this series (17), a medicalization lens can be useful for critically examining the contemporary global health agenda, including what and how issues and problems get prioritised and framed, and what solutions are advanced. The conditions that comprise the priority NCDs are by definition ‘diseases’, and the problem is widely described as a ‘global emergency’ and an ‘epidemic’ – in other words, the NCD problem is already framed in medical terms. So the challenge and opportunity lie in developing and enacting strategies that are more comprehensive than those that individualise the solutions and place healthcare and biomedical interventions central, which could fuel medicalization and limit success.

I trace three features.

## Individualistic expectations

The first concerns the tension between individual versus population-level interventions. That both population-level (such as legislation) and individual-level (such as decreased consumption or use of medicines) strategies are needed to address common risk factors and meet global targets is clearly acknowledged in the agreed political declaration, WHO's comprehensive action plan, and much of the literature including the ‘best buys’ (19). But the recommendations very much lend themselves to the individual level even when they are held up to be ‘population level’. These so-called population strategies or public health actions (16, 20) are often actually expectations of individual and behavioural change, which will have limited success and impact, and deflect attention away from government policies or regulation of industry.

The individualistic approach – classically seen in health promotion, health literacy or mass education strategies – views the cause and thus the responsibility for reducing exposure to risk factors in individuals, and is emblematic of a medicalized framing of health problems. In the medicalization of obesity, for example, healthcare strategies and nutritional intervention reinforce how the matter is one of individual responsibility and broader action amounts to changing the immediate environment so that individuals can make more appropriate choices (21, 22), thus failing to see the broader social and environmental contexts. In these contexts, particularly those that create multiple disadvantages or vulnerabilities, the degree to which individuals have sufficient autonomy to make meaningful behavioural change is very much in doubt.

Where individualistic interventions masquerade as public health strategies is apparent in risk factor modification strategies for NCDs (Box 1). Some have described this reductionism as ‘lifestyle drift’, where policy-makers may start with or promote themselves as recognising the ‘upstream’ social determinants of health – for example, marketing of unhealthy food to children – and then drift ‘downstream’ to rely on strategies to directly change the behaviour of individuals (23, 24). Such a drift is imbued with medical ideology, is often skewed towards acute rather than preventative approaches, and can lead to victim blaming (23). Emphasising such an imperative for behaviour choice, however, obscures the circumstances in which individuals make that choice or indeed have their choices constrained. As Moodie and colleagues have argued, the pricing, availability, marketing and perceptions of costs and benefits strongly influence choice of unhealthy product consumption (25). Similarly, Hunter and Reddy note that dietary risk factors and physical inactivity are only partially determined by individual preferences – and are more so ‘substantially influenced by the manufacturing and marketing practices of the food industry and by the built and social environments that permit or impede physical activity’ (26).

***Box 1.*** So-called Public or Population Health Strategies Can Reduce to Individualism

Even when held up as public health or population-level actions, many NCD strategies are expectations of individual or behavioural change that fail to see individual risk factors within their social and environmental contexts. For example:

- ‘Get moving’ campaigns even when targeted at the whole population fail to view barriers to physical activity as including declining opportunities for movement due to urbanisation, sedentary work and motorised transport, and the built and social environments in which people live and work (26, 27).
- Quit smoking campaigns, even when scaled broadly, often view tobacco exposure as an individual choice not influenced by social norms, health beliefs, economic and employment circumstances and marketing by tobacco companies. Global tobacco control strategies often do not recognise tobacco exposure to be secondary in some environments to indoor air pollution as the major risk factor (27).
- Health promotion interventions aimed to reduce consumption of unhealthy food and alcohol fail to see the context in which choice is constrained: the pricing and availability of products, marketing practices of food and beverage companies, and the liberalisation of trade and commodities of markets that create dietary dependency (25).
- The ‘population-level’ mass education strategies advocated to encourage salt reduction and substitution to lower blood pressure (16, 19) shift the responsibility to individuals for making behavioural changes, rather than on the food companies that produce and profit from highsalt processed foods. This reductionistic shift holds even when those strategies are combined with attempts to regulate the food industry if those measures involve selfregulation or voluntary changes in practice.

Adopting the values of a medicalized approach (28) – immediacy, efficiency and control – the use of ‘quick fix’ or ‘magic bullet’ strategies designed to influence individual choices rather than government policies or the activities of manufacturers is tempting in the short term, but will not have a lasting impact (27), especially as NCDs are often interconnected and require long-term solutions. Even drug interventions like the polypill (a mix of low-dose, low-cost drugs to lower blood pressure and cholesterol plus a folic acid vitamin), which might be more effective than expecting individuals to modify their lifestyle, cannot be assured to scale-up to the population level and achieve health gains (27). As Veerman argues, whether it is salt reduction, increased physical activity or decreased tobacco or harmful alcohol consumption, there is a population-level intervention (such as taxation, legislation, nutritional labelling, marketing restrictions or other policy responses) that will be more effective than an individual approach (29).

Of course, this individualistic framing of the NCD problem and its solutions finds support in industry, not least because a focus on individual behaviour provides cover for the tobacco, food, beverage and alcohol industries uninterested in having their production and marketing of products curtailed through regulation or restrictions, nor the profits that wide consumption ensures. In other words, industry has a stake in medicalization because such a framing of NCDs serves its interests. And industry has had an influential place in the NCD agenda-setting. At the 2011 UN Summit, lobbying by food and beverage companies effectively dampened the discussion and inclusion of actions related to the most cost-effective, fiscal and regulatory interventions (30), and at the 2013 World Health Assembly industry touted the ‘harmful’ effects of taxation and marketing restriction (31). Instead, industry advocates for alternatives in the form of the ineffective, individually targeted information and educational approaches that place responsibility for decisions on the individual (25). The recommendations and agreements about reaching the smoking target stand in contrast: stronger implementation of the Framework Convention on Tobacco Control, which targets the industry through taxation and restriction of products, although here too there remain substantial challenges: less than 10% of the world is covered by the key tobacco control policies agreed by more than 170 member states (32).

Clearly, individualistic approaches do not address the root causes of the NCD problem, which not only include industrial activity but also elements of the global political economy that give rise to the problem in the first place. Sometimes called the ‘causes of the causes of the causes’ (27, 33), many commentators argue that these broader societal factors can be tackled only by enacting policies that address structural drivers, especially those that produce poor nutrition: liberalisation of food trade; the escalation of foreign investment by transnational food companies; the rapid spread of cheap, ultra-processed food that affect the availability, affordability and acceptability of what people eat; and deregulation of commodities markets that cause food price volatility and dietary dependency (34). Other needed broader social policy interventions to address root causes of NCDs relate to urban design, transportation policies and social spending to provide protection against unemployment, housing insecurity, old age and disability, among others, all of which reflect the ‘intersectoral, whole of government’ approach advanced by many NCD advocates (5, 16, 20). Such interventions, which would broaden the framing of the NCD problem, require political will (29). Unfortunately, the prevailing culture of health and social policy in LMICs – donor-driven, dependent upon external financing with short-term and results-based thinking and focused on economic growth – does not lend itself to structural interventions targeted at the root causes (35, 36).

## More doctors, medicine and markets

The second issue contributing to the medicalization of the NCD agenda is the provision of enhanced roles for medical professionals and affiliated health workers working under a medical model of care, and the reinforcement of the importance of medical products and monitoring. Placing access to medicines central in the agenda for addressing ‘the global emergency of NCDs’ (37) supports the prominence and expansion of healthcare solutions in responding to the ‘crisis’, both curative and preventative. For example, the priority intervention of multidrug treatment for primary and secondary prevention of cardiac events (16, 19, 38), along with ‘best buys’ of screening for cervical cancer and hepatitis B immunisation to prevent liver cancer (19), create more medical requirements and settings for dealing with the NCD problem: new methods of task shifting, resourcing and delivering these drug regimens, and new needs for medical support in the form of evidence-based guidelines, treatment protocols, clinical training and standards, physician back-up and oversight of roll-out. In NCD prevention and management programmes generally, physicians in particular are said to need to become more involved (16), and others have emphasised the responsibility of doctors in enacting priority NCD strategies: to encourage people to make healthy living choices, promote health literacy and provide health services focused on early detection and cost-effective management of NCDs and their risk factors (26).

Much like how medical professional involvement and control over harm reduction contributed to the medicalization of tobacco use (39), defining and developing a strategy that provides new opportunities or central roles for medical professionals support the process of medicalization of NCDs. On the one hand, task shifting and use of community health workers to provide access to medicines, blood glucose and cholesterol monitoring, and a variety of types of NCD screening might be seen to de-medicalize the process by drawing upon less skilled or non-professionalised staff. But on the other hand, it can be seen as simply mobilising more and different sets of healthcare workers to apply individualised strategies and biomedical approaches and understandings. Even when positioning strategies as integrated multi-intervention packages to tackle a complex problem, such approaches – if dominated by medical models of care – can leave underlying causes unaddressed: Sanders reports how UNICEF's GOBI package for child survival was presented as a cost-effective and efficient form of primary healthcare, but that ‘health interventions remained resolutely within medical control where simple evidence-based results could be observed’, providing governments and health workers a way to avoid the social, economic and political causes of poor health (35). Dominance of doctors in the response to NCDs will also undoubtedly increase costs.

## NGO voices drowned out by industry

Third, there is a relative lack of an engaged civil society and few non-governmental organization (NGO) voices. The predominance of the professional view in the framing of the NCDs problem, to the exclusion of the patient view, has been noted (40), and it seems clear that this is one part of a bigger problem in the NCD agenda-setting: the lack of community and advocate participation that has in previous cases countered attempts to medicalize human experience and ill health. For example, as the HIV crisis grew in the 1980s and 1990s, well-organised groups of activists resisted attempts to medicalize the response and pushed for collective solutions (41); for decades, women's health advocates have fought the medicalization of women's bodies, contesting medical definitions of ‘normal’ pregnancy, birth and sexuality, and resisting ‘treatments’ involving technology and drugs that limit women's choices (42). In contrast, in the case of NCDs, the nature of its ‘civil society’ appears to reinforce rather than challenge the medicalization of NCDs.

The lack of a broad, engaged NGO community in the NCD agenda-setting is said to be one reason why the industry view is so strong (30); others have observed how the rise of the ‘corporate NGO’ now dominates civil society participation in the NCD space, effectively crowding out more progressive, independent, community-based organisations (David Sanders, personal communication). At the 2011 UN civil society hearings – the chief opportunity for advocates to shape the final UN political declaration – the representatives of civil society were not independent NGOs but representatives from food (including the International Food and Beverage Alliance) and alcohol (including Anheuser-Busch, SABMiller, and the Global Alcohol Producers group) industries (30). Medical organisations have also been champions of the NCD ‘fight’, in particular, the non-governmental NCD Alliance that was founded by four major disease federations – the World Heart Federation, the International Diabetes Federation, the Union for International Cancer Control and the International Union against Tuberculosis and Lung Disease – and now has over 900 member organisations. The most prominent, organised and visible of all advocacy groups, the NCD Alliance appears particularly keen to promote interventions that restrict tobacco, alcohol and food industry activities (including taxation and exclusion of the tobacco industry from agenda-setting), while also actively promoting targets that would widen the use of pharmaceutical products (43, 44). Critics (45) have highlighted the funding base of the NCD Alliance, comprised of major pharmaceutical and medical technology companies, all of whom would benefit enormously from expanded mandates for cancer, cardiovascular and risk factor screening and treatment.

Indeed, the involvement and the role of the pharmaceutical industry are means to reinforcing NCDs as a ‘medical kind of problem’. Medicalized accounts of NCD solutions sit very comfortably within the space in which drugs, devices and other physical entities predominate, and so it is no wonder that the industrial sector has interests in developing this particular aspect of the NCD agenda. The pharmaceutical industry contributes to a medicalized NCDs approach with efforts to expand boundaries of ill health by defining diagnostic categories of pre-diabetes and pre-hypertension (46) and benefits when risk factor thresholds change to create more patients for drugs, as in the recent cholesterol and statins guidelines (47). While some commentators seek to reassure critics that treatment areas such as the secondary prevention of cardiac disease or diabetes can depend upon the use of low-cost, off-patent drugs and thus offer pharmaceutical companies no financial gain that could lead to nefarious intentions in the NCD campaign, industry reports suggest otherwise. According to market research firms (48, 49), total global pharmaceutical sales was US$1.08 trillion in 2011, with sales in ‘growth markets’ (such as Brazil, Russia, India and China) rising by 22.6% and expected to double by 2020 to US$300 billion annually. In the growth markets, ‘most of the projected increase in pharmaceutical sales over the next decade is expected to come from generics rather than patented products’ (48). Indeed, as Bollyky argues, ‘the multinational pharmaceutical industry has staked its future on [non-communicable] diseases and emerging markets’, and will oppose flexible intellectual policy policies of the likes that provided wider access to HIV/AIDS drugs (50). Furthermore, there remains a market for new cardiovascular and cancer drugs. But it is in vaccines (especially prophylactic ones) ‘where industry will find its “El Dorado”’ (gold), according to PriceWaterhouseCoopers (PWC), which reports that vaccines for cancer, cardiovascular disease, diabetes and obesity are already in clinical development (48, 51). Device or technology companies, too, may find comparable interests in a medicalized solution and in markets for screening and monitoring tests.

Many commentators in the NCD field are appropriately sceptical as to whether healthcare solutions alone can address the NCD problem. In reflecting the technological values of medicalization, drug and device (and surgical) options offer efficiency that modifying physical activity, taxing unhealthy foods, banning sales of tobacco or restricting food marketing cannot. But as noted, these medicalized ‘magic bullets’ rarely produce a lasting impact (27, 33). And the consequences of medical care can include, as other analyses have demonstrated (21, 52, 53), creating sick patients, overdiagnosis, adverse effects of treatment, stigma for patients and major costs, although the latter are argued to be justified by the costs-savings of treating long-term consequences of diseases (16). Technologies, in turn, promote the idea of the NCD patient as amenable to manipulation and repair, and detract from social contexts that give rise to the vulnerability and risk in the first place. Widening the frame would acknowledge the long-standing recognition that social and political determinants, contexts and drivers are at the heart of the NCD problem, and in doing so will challenge medicalization of the NCD agenda.

## How can the medicalization of the NCD problem be challenged?

First, it would seem necessary to tilt the NCDs agenda's balance of individual and societal approaches, in order to genuinely achieve the multi-sector, whole-of-society response that is recommended in action plans. As Stucker and colleagues argue, greater health gains will be achieved by acting on societal determinants and would ‘improve longevity and quality of life much more than giving people access to all the best medications in the world’ (27). Indeed, the recent Lancet Commission on Investing in Health report affirms the need for a combination of inexpensive population-based and clinical interventions, strengthening of health systems and fiscal policies such as taxation, regulation, subsidies and other legislation to curtail the health and economic effects of NCDs (54). To truly recognise NCDs as diseases of poverty (5) will require action on the societal determinants of health that create inequities – avoiding the drift to individualistic strategies when ones that tackle the structural drivers are necessary. This will require a substantial reframing of the ways to reach the global targets than is currently presented, but as Alleyne and colleagues argue (5), equity must be incorporated into progress, otherwise ‘the poorest people could be further marginalised and disparities might continue to grow’. Acting on upstream determinants is more equitable than downstream determinants (55), but this requires strong action and political will (29, 34), neither of which are simple. Undoubtedly, many NCD proponents would argue that considerable progress can be made with existing knowledge and strategies, even as the global health community struggles to understand and target the ‘causes of the causes’ (16, 54).

Nonetheless, the NCD agenda needs to be more politicised. How can this be achieved? One way would be to expand research efforts on understanding the broader societal and political contexts. Clearly, the medicalization of NCDs provides opportunities for profiteering and a platform from which to expand the markets of NCD patients; outcomes of corporate behaviour on health that demand further critique and research (25, 36). That the globalised political economy enables this through neoliberalist policies, free trade and the concentration of power and influence within medical elites and multinational companies (56–58) further emphasises the need for research and analysis to encompass the political context of NCDs. The barriers to doing so will not be inconsiderable. As Navarro argues, research into the politics in public health is rare due to the disinclination of researchers to tackle the ‘dirty issues’ that may disrupt the neutral agendas of public funders (57, 58). In addition, this type of research will require confronting industry, which D'Ambruoso (59) describes in relation to the need for the post-2015 agenda to examine the structural contexts of health inequities, as having ‘strong political interests related to market freedom and the pursuit of profit for growth that are pursued and enacted through aggressively lobbying and legislative influence’. Uncovering and understanding the societal, political and ideological determinants of NCD strategies can help craft more effective responses and change (36). However, ‘interventions will never be simple to do when vested interests stand to lose profits from them’ (36), a point recently reaffirmed by the Lancet Commission on Investing in Health that acknowledged the difficulties in addressing societal determinants of health where ‘complex and entrenched political obstacles exist’, and where effects ‘will not be realised for a long period’ (54).

Second, and similarly, advocates must resist industry influence on the framing of the NCD debate that reinforces individualistic choices. The private sector clearly has a responsibility in the NCD problem and sees a role for itself that includes promoting healthy workplaces, developing new products and new markets, and assuming corporate social and environmental responsibility (60). As Hogerzeil and colleagues argue (38), the pharmaceutical industry can also facilitate access to low-cost medications. But many players in the industry sector clearly should not have a role in the agenda-setting because some companies themselves are the ‘major drivers of global epidemics of NCDs’ through their sale and promotion of tobacco, alcohol and ultra-processed food and drinks (25). Furthermore, by reinforcing individual choice and responsibility that deflects broader political reforms (which industry labels ‘nanny-state’), such industry contributions to the agenda-setting obscure their broader strategies for undermining public health policies and programmes: to bias research findings, co-opt policy-makers and health professionals, lobby politicians and governments to oppose public regulation, and encourage votes to oppose regulation of their activities, say Moodie and colleagues (25). Based on a comprehensive review, they conclude that there is no evidence for the effectiveness or safety of industry self-regulation nor public–private partnerships, and instead that the best mechanisms to prevent harm caused by the tobacco, alcohol, food and drink industries in the NCD pandemic are public regulation and market intervention including legislation, taxation, pricing, bans and restriction of advertising and sponsorships (25).

Rather than partnering with industry, NCD advocate efforts should be focused on strengthening social movements and the NGO space to counter the industrial and the professional views that currently dominate the medicalized framing of NCDs. Incorporating the patient view and the view of civil society organisations through participatory research methodologies that work for social and systemic change (56) may reveal ways to de-professionalise the response to NCDs and the perceived benefits to individuals of medicalization or de-medicalization (61); although promoting scenarios whereby patient groups merely advocate for the latest drug should be avoided in so far as it would reinforce the individualistic view. Any growth in the NGO space, including the continued work of the influential NCD Alliance funded by disease federations with major pharmaceutical industry backing, should be accompanied by transparency of financial sources, management of real or perceived conflicts of interest and independence from companies that have a financial stake in the outcomes of NCD agendas and international commitments. Some commentators advocate partnering with environmental activists (5), among others, which might stimulate the social and community perspectives needed to help recast the NCD issues as collective problems in need of social solutions. These actions can include building social networks, reframing debates from individual responsibility to collective focus on social inequalities, working collectively to secure the resources for healthy living and supporting regulation and litigation when necessary (27), thus supporting the integrated, comprehensive response of community level action and political will and investment that can overcome the global problem of NCDs.
